# Markerless motion capture systems as training device in neurological rehabilitation: a systematic review of their use, application, target population and efficacy

**DOI:** 10.1186/s12984-017-0270-x

**Published:** 2017-06-24

**Authors:** Els Knippenberg, Jonas Verbrugghe, Ilse Lamers, Steven Palmaers, Annick Timmermans, Annemie Spooren

**Affiliations:** 1 PXL University College, Guffenslaan 39, 3500 Hasselt, Belgium; 2 REVAL - Rehabilitation Research Center, BIOMED - Biomedical Research Institute, Faculty of Medicine and Life Sciences, Hasselt University, Hasselt, Belgium

**Keywords:** Motion capture system, Nervous system diseases, Rehabilitation, Systematic review

## Abstract

**Background:**

Client-centred task-oriented training is important in neurological rehabilitation but is time consuming and costly in clinical practice. The use of technology, especially motion capture systems (MCS) which are low cost and easy to apply in clinical practice, may be used to support this kind of training, but knowledge and evidence of their use for training is scarce. The present review aims to investigate 1) which motion capture systems are used as training devices in neurological rehabilitation, 2) how they are applied, 3) in which target population, 4) what the content of the training and 5) efficacy of training with MCS is.

**Methods:**

A computerised systematic literature review was conducted in four databases (PubMed, Cinahl, Cochrane Database and IEEE). The following MeSH terms and key words were used: Motion, Movement, Detection, Capture, Kinect, Rehabilitation, Nervous System Diseases, Multiple Sclerosis, Stroke, Spinal Cord, Parkinson Disease, Cerebral Palsy and Traumatic Brain Injury. The Van Tulder’s Quality assessment was used to score the methodological quality of the selected studies. The descriptive analysis is reported by MCS, target population, training parameters and training efficacy.

**Results:**

Eighteen studies were selected (mean Van Tulder score = 8.06 ± 3.67). Based on methodological quality, six studies were selected for analysis of training efficacy. Most commonly used MCS was Microsoft Kinect, training was mostly conducted in upper limb stroke rehabilitation. Training programs varied in intensity, frequency and content. None of the studies reported an individualised training program based on client-centred approach.

**Conclusion:**

Motion capture systems are training devices with potential in neurological rehabilitation to increase the motivation during training and may assist improvement on one or more International Classification of Functioning, Disability and Health (ICF) levels. Although client-centred task-oriented training is important in neurological rehabilitation, the client-centred approach was not included. Future technological developments should take up the challenge to combine MCS with the principles of a client-centred task-oriented approach and prove efficacy using randomised controlled trials with long-term follow-up.

**Trial registration:**

Prospero registration number 42016035582.

**Electronic supplementary material:**

The online version of this article (doi:10.1186/s12984-017-0270-x) contains supplementary material, which is available to authorized users.

## Background

People with central nervous system diseases such as multiple sclerosis (MS), stroke and spinal cord injury (SCI), demonstrate among others loss of motor and sensory function in the upper and lower limbs. Due to motor impairment in upper limbs, the performance of activities of daily life, sports and leisure activities is affected. Motor impairment in the lower limbs, affects mobility in general and balance control during reaching movement. The impairments of both upper and lower limbs reduce functional independence and thus the quality of life of the individual [1–6]. Exercise therapy has proven to improve impairments [7–9], therefore rehabilitation is very important for these patients.

In neurological rehabilitation, training should be challenging, repetitive, task-specific, motivating, salient and intensive to activate neuroplasticity [4]. Moreover, studies have shown the importance and benefits of client-centred task-oriented rehabilitation [10, 11]. The concept of client-centredness not only incorporates patient’s wishes and needs in their rehabilitation, but also actively involves the patient in selecting goals for their own rehabilitation process. Definitions of task-oriented training are still very diverse, but it incorporates that training is directed to a specific, functional, task [10, 12]. Task-oriented training has been proven to be effective in arm-hand skilled performance in stroke patients [12, 13], spinal cord [10] and MS [14]. Spooren et al. [14] demonstrated the importance of specificity of training and inclusion of ‘client-centred training’ and ‘exercise progression’. Timmermans et al. [12] concluded that training components, such as random and distributed practice, together with feedback and clear functional goals, should be incorporated in order to enhance the outcomes of task-oriented training. Despite the advantages of a client-centred task-oriented approach with regard to training outcome and motor learning, this approach requires individualised training schemes and guidance of a therapist. Therefore a client-centred and task-oriented approach is more time consuming and costly for therapists and rehabilitation centres. Hence a new approach is needed where client-centred task-oriented rehabilitation can be administered without extra costs and effort of therapists.

Technology-based rehabilitation systems such as robotics and virtual reality (VR) are promising and may be able to deliver a client-centred task-oriented rehabilitation without extra costs and effort of therapists. Several studies addressed the positive effects of robotics and VR systems as additional therapy in neurological rehabilitation [4, 15–19]. Robotics have shown positive effects such as the enhancement of function and activity of affected limb and increased motivation, but the costs of the devices is high [3, 20]. In addition, the devices are often uncomfortable as the user needs to wear apparatuses on the body and patients have difficulty using such devices [3]. Although a few studies include some aspects of a client-centred approach in robotic rehabilitation, it remains very difficult to incorporate a full client-centred approach because of the wide variety of choices that can be made (e.g. difficult to select individual parameters, specific movements or activities, to use objects, etc. [19, 20]. VR, on the other hand, is a computer-based technology that allows users to interact with simulated environments and receive feedback on performance. VR also stimulates the increase of intensity of movements, therefore it may facilitate motor learning and neuroplasticity through repetition and increased intensity during task-oriented training [2–4]. Compared to the traditional methods used in motor rehabilitation of patients with neurological disorders, VR has some advantages: 1) patients can perform different rehabilitation exercises, recreated in a virtual way (i.e. virtual rehabilitation exercises), 2) VR can set up the features of the exercises, control their performance and acquire relevant data from the patient’s performance, and 3) VR can facilitate the interaction between patient and system through a variety of available devices, such as MIT-Manus, RemoviEM, etc. [21, 22]. Non-immersive video games are also a form of VR. They are developed by the entertainment industry for healthy population and home use making it less costly and more acceptable. Markerless (i.e. without markers or sensors on the body) motion capture systems (MCS) such as Nintendo Wii and Playstation Move, make use of non-immersive video games and have been used in VR rehabilitation. Studies showed an increase in motivation for rehabilitation as well as improvement in motor function and correctness of movement after training. Although the results are positive, these commercially available MCS systems with VR have to date limited utility in rehabilitation for impaired populations [1, 3, 4]: the standard games are too difficult or progress too quickly, they do not provide impairment-focused training (e.g. no treatment towards flexion synergies), and do not specifically address independent home usability and safety [1]. Only a few studies have looked into customising Kinect games for stroke, but no specific focus was payed to the coordination patterns which are important in stroke recovery, reducing compensation strategies, or usability and safety for independent home use [1, 23]. At present, validity and accuracy of the Microsoft Kinect in clinical assessment is strong regarding postural control and standing balance [24, 25]. The reproducibility of Kinect when analysing planar motions is similar to traditional marker-based stereophotogrammetry systems [26]. Although there is an increasing number of studies involving markerless motion capture systems in neurological rehabilitation, the knowledge and evidence of training content and training efficacy with Kinect or other markerless motion capture systems is scarce [24, 27].

Because little is known about the various markerless MCS used in neurological rehabilitation, their implementation in rehabilitation training, and effectiveness as a potential device in client-centred task-oriented training, the present study aims to investigate 1) which (markerless) motion capture systems are used as training devices in neurological rehabilitation, 2) how they are applied, 3) in which target population, 4) what the content of the training is and 5) what the efficacy of training with MCS is.

## Methods

### Search strategy

A computerised search was conducted in PubMed, Cinahl, Cochrane Database and IEEE. Studies were collected up to December 2016.

The following Medical Subject Headings (MeSH) and key words were used: (“Motion”[Mesh] OR “Movement”[Mesh] OR motion[Title/Abstract] OR movement[Title/Abstract] AND detection[Title/Abstract] OR capture[Title/Abstract] OR kinect[Title/Abstract]) AND (“Rehabilitation”[Mesh] OR rehabilitation[Title/Abstract]) AND (nervous system diseases[MeSH Terms] OR nervous system diseases[Title/Abstract] OR multiple sclerosis[MeSH Terms] OR multiple sclerosis[Title/Abstract] OR stroke[MeSH Terms] OR stroke[Title/Abstract] OR spinal cord[MeSH Terms] OR spinal cord[Title/Abstract] OR parkinson disease[MeSH Terms] OR parkinson disease[Title/Abstract] OR brain injuries, traumatic[MeSH Terms] OR brain injuries, traumatic[Title/Abstract] OR cerebral palsy[MeSH Terms] OR cerebral palsy[Title/Abstract]) NOT (“Eye”[Mesh] OR eye[Title/Abstract] OR “Speech”[Mesh] OR speech[Title/Abstract]).

Two review authors (EK & JV) conducted the search and the inclusion of the articles.

### Inclusion criteria

Studies were included when persons with nervous system diseases, such as MS, stroke, SCI, cerebral palsy, etc., were involved in an intervention study or trial in which a markerless MCS was used in the rehabilitation program to improve the upper or lower limb function or balance control. All studies published in English, French, German and Dutch were included. All quantitative study designs, except systematic reviews were included in this review.

### Exclusion criteria

Studies on healthy subjects, children or animals were excluded. Training with robotics or exoskeletons were also excluded. Studies involved with eye or speech motion capture, gait/fall capture, movement intent and motion assessment were not eligible for further analyses.

### Methodological assessment

Two review authors (EK and JV) independently assessed the methodological quality of all selected studies with the Van Tulder’s Quality assessment scale [28]. This scale scores the internal validity (score 0–11), descriptive criteria (score 0–6) and the statistical criteria (score 0–2) of randomized controlled trials (RCT) and controlled clinical trials (CCT), but was also used to evaluate the quality of studies with another study design. The items “Was the care provider blinded for the intervention” and “Was the patient blinded to the intervention” of the internal validity score were considered to be not applicable as care providers and patients are aware of the training they provide or receive. The interrater reliability of the individual items was tested using the Cohen’s Kappa [29]. The total Van Tulder score (0–17) was calculated after any disagreements were discussed and resolved.

### Data extraction

The type of motion capture system, the patient group and training components used in all selected studies were described to answer the first two aims of this systematic review. The following training components were extracted: the ICF training level, trained body part (upper limb, lower limb or full body), format of content (exercise, game or task), real-object or VR, training components (e.g. weeks, frequency, etc.), feedback, use of client-centred approach and training outcome. In order to describe the training outcome of the studies (third aim), only studies with a good methodological quality (i.e. score of nine or higher) were incorporated, nine being the quality cut-off point of 50% (on 17 items) suggested by Van Tulder [28].

## Results

Additional file 1: Figure S1 summarizes the stages of the article search and the inclusion/exclusion process. A total of 638 articles were identified and after exclusion by screening on title/abstract (*n* = 549), 66 articles were selected for further evaluation. Fifty articles were further excluded by screening full-text and two references were added. At the end, 18 papers were selected and analysed.

### Methodological quality of the included articles

Table 1 presents the Van Tulder score of the 18 selected papers including the score of internal validity, the descriptive score, statistical score and total score.

**Table 1:** Van Tulder score of each selected study, system used and subjects

Reference	Design	Van Tulder score				System	Patient (n)
		IV	DC	SC	Total		
Bao 2013 [34]	Case series	2	4	2	8	Kinect	Subacute stroke (5)
Brokaw 2014 [1]	Case study	1	1	1	3	Kinect	Chronic stroke (1)
Chang 2011 [36]	Case study	1	1	1	3	Kinect	Dementia and brain injury (2)
Jaume-i-capo 2014 [6]	Case series	1	4	2	7	Kinect	Cerebral Palsy (8)
Lee 2013 [31]	CCT	3	3	2	8	Kinect	Chronic stroke (14)
Levin 2012 [2]	RCT	8	5	2	15	GestureXtreme	Subacute stroke (12)
Lloréns 2012 [5]	Case series	1	3	2	6	Kinect	Chronic stroke (15)
Lloréns 2015 [32]	RCT	7	5	2	14	Kinect	Chronic stroke (20)
Lloréns 2015 [33]	RCT	6	5	2	13	Kinect	Chronic stroke (30)
Lozano-Quilis 2014 [21]	RCT	5	5	2	12	Kinect	MS (11)
Palacios-Navarro 2015 [39]	Case series	3	3	2	8	Kinect	PD (7)
Pastor 2012 [23]	Case study	2	1	1	4	Kinect	Chronic stroke (1)
Pompeu 2014 [37]	Case series	3	2	2	7	Kinect	PD (7)
Shiri 2012 [35]	Case series	3	4	2	9	Motion capture platform	(Sub)acute stroke (6)
Sin 2013 [3]	RCT	4	5	2	11	Kinect	Chronic stroke (35)
Summa 2015 [41]	Case series	2	1	1	4	Kinect	Chronic stroke and TBI (4)
Summa 2015 [40]	Case series	2	3	2	7	Kinect	PD (7)
Ustinova 2013 [38]	Case series	2	2	2	6	Kinect	Chronic, mild-to-moderate TBI (9)

*IV* internal validity, *DC* descriptive criteria, *SC* statistical criteria, *CCT* controlled clinical trial, *RCT* randomized controlled trial, *MS* multiple sclerosis, *PD* Parkinson’s disease, *TBI* traumatic brain injury

There was disagreement on nine of the 306 items on the Van Tulder score, resulting in a mean Cohen’s kappa score of 0.91 between the two raters, which is considered an excellent agreement [30].

After consensus on all items, the mean Van Tulder score was 8.06 ± 3.67 (mean ± standard deviation (SD)), with a mean internal validity score of 3.11 ± 2.11 (out of 9), a mean descriptive score of 3.16 ± 1.54 (out of 6) and a mean statistical score of 1.78 ± 0.43 (out of 2). Overall it can be stated that the internal validity and descriptive score were low. This may be due to the use of the Van Tulder scale for all designs while the scale is developed especially for RCTs and CCTs.

Six out of 18 studies included a control group, consisting of five RCTs and one CCT [31]. Although five of the 18 included studies were RCT’s [2, 3, 21, 32, 33], only one study was single blinded [21]. No studies were double blinded. All studies had short-term follow-up measurements performed, while only two had long-term follow-up measurements performed [34, 35].

The six selected studies with a Van Tulder score of 9 or higher will be used to describe training efficacy [28]. These six studies had a mean Van Tulder score of 12.33 ± 2.16 (mean ± SD).

#### Type of motion capture system

Three different types of MCS were used, as shown in Table 1. Sixteen studies involved training with Microsoft Kinect system [1, 3, 5, 6, 21, 23, 31–34, 36–41], one study used Gesture Xtreme (which was not described in detail in the original publication) [2] and one study used a motion capture platform but did not further specify the system [35].

First and most frequently used MCS is the Microsoft Kinect. The Microsoft Kinect is an infrared motion capture device used for interactive computer games, intended for the Xbox 360 game console. The device enables users to control and interact with the virtual reality environment via infrared camera and depth sensor, without the need for a remote controller. Movement of the user is provided in full-body 3D motion detection capabilities and gesture recognition, captured in real time and feedback is provided immediately. It is commercially available at low cost [3, 34, 36].

The second system described, is the Gesture Xtreme. This is a virtual gaming system that uses patented video gesture control software and immersive technology. The system transports your body into a computer generated landscape. Human body gestures make it possible to interact with the virtual world in real time [2, 42].

Third, Shiri et al. [35] describe their system as a motion capture platform which is operated with a standard laptop and a low-cost webcam. Patients have to wear a white robe and black sleeveless vest against a blue background for the system to be able to recognise the person. This novel VR system replaces the impaired arm of the patient by a virtual one. The virtual arm is controlled by the patients who use a mouse, trackball or joystick to manipulate the virtual arm. The system integrates self-face viewing online with mirror visual feedback, but the name of the system is not further specified.

#### Target population

Different target populations with neurological problems were involved in the studies. However, the majority of the studies (*n* = 11) included persons with stroke [1–3, 5, 23, 31–35, 41], of which eight with chronic stroke [1, 3, 5, 23, 31–33, 41]. Other patient groups where only described in one to three studies and included dementia and brain injury [36], cerebral palsy [6], MS [21], Parkinson’s disease [37, 39, 40], traumatic brain injury [38].

Sample size of the studies varied between one (case study) [1, 23] and 35 (RCT) [3] with a mean age between 32.3 years [38] and 76.4 years [31].

Regarding patient group and type of MCS, half of the selected studies used the Kinect with stroke patients [1, 3, 5, 23, 31–34, 41].

#### Training content

As shown in Table 2, there is a wide variety between the training content of the included studies (i.e. ICF training level, the body part trained, content of the training, exercise, game or task, use of real- and/or virtual object, the feedback mechanism used, and training intensity).

**Table 2:** Training parameters of all selected studies

Reference	ICF level	Body part	Content	Format	Real-object or VR	Feedback	Weeks or sessions	Frequency
Bao 2013 [34]	A	UL	Fruit Ninja: Slicing fruit	Games	VR	Visual	3 weeks	5 × 1 h/week
Brokaw 2014 [1]	A	UL	Functional reach and shoulder abduction with elbow extension	Games	VR	Visual	4 weeks	5 × 1 h/week
Chang 2011 [36]	A + P	UL	Preparing pizza	Tasks	VR	Visual and auditory	Unknown	2 sessions/day
Jaume-i-capo 2014 [6]	F	FB	Standing and reaching movements	Exercises	VR	Visual and auditory	24 weeks	1 × 20 min/week
Lee 2013 [31]	A	UL	Kinect sports and Kinect adventures	Games	VR	Not mentioned	6 weeks	3 × 30 min/week
Levin 2012 [2]	A + P	UL	Goal-directed reaching tasks	Games	VR	Visual	3 weeks	3 × 45 min/week
Lloréns 2012 [5]	A	LL	Stepping exercises for balance control	Exercises	VR	Not mentioned	20 sessions	3–5 × 45 min/week
Lloréns 2015 [32]	A	LL	Stepping exercises for balance control	Exercises	VR	Not mentioned	4 weeks	5 × 1 h/week
Lloréns 2015 [33]	A	LL	Stepping exercises for balance control	Exercises	VR	Not mentioned	20 sessions	3 × 45 min/week
Lozano-Quilis 2014 [21]	F + A	LL	Balance and weight	Exercises	VR	Visual	10 weeks	1 × 1 h/week
Palacios-Navarro 2015 [39]	A	LL	Lateral leg movement	Exercises	VR	Visual	5 weeks	4 × 30 min/week
Pastor 2012 [23]	F	UL	Reaching target	Exercises	VR	Visual	2 weeks	5 × 10-20 min/day
Pompeu 2014 [37]	A	FB	Kinect adventures	Games	VR	Visual and auditory	14 sessions	3 × 1 h/week
Shiri 2012 [35]	A	UL	Arm movements	Games	VR	Visual	10 sessions	2–3 × 45 min/week
Sin 2013 [3]	A	UL	Kinect sports and Kinect adventures	Games	VR	Visual and auditory	6 weeks	3 × 1 h/week
Summa 2015 [41]	F	LL	Reaching target	Exercises	VR	Visual	6–10 sessions	1 h
Summa 2015 [40]	A	FB	Reaching target	Exercises	VR	Visual	10 sessions	2 × 40 min/week
Ustinova 2013 [38]	F + A	FB	Coordination and postural control	Exercises	Mixed	Visual and auditory	15 session	2–4 × 50-55 min/week

*ICF* International Classification of Functioning, Disability and Health, *F* function, *A* activity, *P* participation, *UL* Upper limb, *LL* Lower limb, *FB* Full body, *VR* Virtual reality

Most studies (*n* = 11) reported a training specifically on the ICF activity level, it was remarkable that in two studies the training program was targeted towards the ICF activity and participation level [2, 36].

Eight studies focused on upper limb training [1–3, 23, 31, 34–36], while six studies concentrated on lower limb training [5, 21, 32, 33, 39, 41] and only four studies trained the full body [6, 37, 38, 40].

The study of Pastor et al. [23] was the only study that provided an upper limb training on the ICF body functions and structures level. In this study a game was developed where the patient needs to select images that randomly appear in a cell of a 2 × 2 to 6 × 6 grid on the screen, depending on the game. Selecting an image is done by locating the cursor inside the appropriate cell by moving the hand. The images used, such as furniture or transportation means, differ every session, but is not changed in accordance to the patient’s interest [23]. The two studies that offered upper limb training on ICF activity and participation level included performing the vocational task of preparing pizza and goal-directed reaching tasks in a virtual supermarket [2, 36]. The remaining five studies performing upper limb training, focused on the ICF activity level [1, 3, 31, 34, 35], mainly performing standard Kinect games [3, 31, 34]. The four studies who trained the whole body, focused on ICF function, activity or the combination of function and activity level [6, 37, 38, 40]. Four out of six studies that trained the lower limbs, focused on activity level by performing stepping exercises for balance control [5, 32, 33] and lateral leg movement [39]. One other study on lower limbs, trained on function and activity level and performed balance and weight exercises [21]. The last study on lower limb focused on ICF functional level by performing pelvis movements [41].

Training content included tasks [36], standardised exercises [5, 6, 21, 23, 32, 33, 38–41] or games [1–3, 31, 34, 35, 37]. Four out of six studies used the commercially available games such as Fruit Ninja [34] or Kinect Sports and/or Kinect Adventures [3, 31, 37] while only two studies took the interest of the patients into account by using visual targets or games, based on the patients’ interest [6, 31]. In the study of Lee et al. [31], the patients had to choose one Kinect Sports game (i.e. Boxing or Bowling) and one Kinect Adventures game (i.e. Rally Ball, 20,000 leaks or Space Pop). Jaume-i-Capo et al. [6] used targets on the screen that could easily be changed into images that are of interest for each user, e.g. football club images. In only one study patients were given the choice of using a real object during training [38].

None of the studies included a client-centred task-oriented approach, such as the Canadian Occupational Performance Measure (COPM). All studies used standardised exercises or games, with respect to therapeutic goals and focused on the impaired body part or functionality, but never involving the patient in the process. Only two studies take patient’s interest into account by using images of their interests [6] or give the patient a choice in which game to play [31].

With regard to feedback during and/or after training, most studies (*n* = 9) only used visual feedback [1, 2, 21, 23, 34, 35, 39–41]. Five studies provided a combination of visual and auditory feedback [3, 6, 36–38] and four studies did not mention their type of feedback [5, 31–33].

The diversity in the training duration and intensity was large over the different studies ranging from only two [23] to 24 weeks of training [6], with a frequency of one to five times a week and sessions of 20 min to 1 h a day. It should be noted that the study with the longest training duration (i.e. 24 weeks), offered the lowest intensity of only once a week for 20 min [6].

#### Training efficacy

An overview of the selected studies (Tulder score ≥ 9), their intervention and their mean outcome is presented in Table 3 for upper and lower limb.

**Table 3:** Training outcome in upper and lower limb studies

Reference	Body part	Design	Intervention				Clinical outcome measures		Results (ICF level)	
			Subjects (n)	ICF level	Weeks or sessions	Frequency	ICF level	Motor outcome	Within group	Between group
Levin 2012 [2]	UL	RCT	Subacute stroke (12)	A + P	3 weeks	3 × 45 min/week	F + A	FMA upper extremity, CSI, RPSS, BBT, WMFT, MAL, patients comments	F^ES^, A^ES^	Stronger effect of training on WMFT in VR group at post-test compared to conventional group.
Shiri 2012 [35]	UL	Case series	(Sub)acute stroke (6)	A	10 sessions, 4 weeks	2–3 × 45 min/week	F + A + P	FMA, WFMT, MAL, DY, BBT, VAS Pain, SF-36	F*, A*, P^£^	NA
Sin 2013 [3]	UL	RCT	Chronic stroke (40)	A	6 weeks	3 × 1 h/week	F + A	AROM upper extremity, FMA, BBT	F*, A*	F*, A*
Lloréns 2015 [32]	LL	RCT	Chronic stroke (20)	A	4 weeks	5 × 1 h/week	A	BBS, POMAb, POMAg, BBA, 10MWT; Short Feedback Questionnaire	A*	A*
Lloréns 2015 [33]	LL	RCT	Chronic stroke (30)	A	20 sessions	3 × 45 min/week	A	BBS, POMAb, POMAg, BBA, SUS, IMI, cost	A*	No significance found
Lozano-Quilis 2014 [21]	LL	RCT	MS (11)	F + A	10 weeks	1 × 1 h/week	F + A	BBS, POMAb, SLB, 10MWT, TUG, SEQ	F*, A*	F*, A*

*UL* Upper limb, *LL* Lower limb, *RCT* randomized controlled trial, *ICF* International Classification of Functioning, Disability and Health, *F* function, *A* activity, *P* participation, *WMFT* Wolf Motor Function Test, *FMA* Fugl Meyer Assessment, *MAL* Motor Activity Log, *AROM* Active Range of Motion, *BBT* Box and Blocks Test, *CSI* Composite Spasticity Index, *RPSS* Reaching Performance Scale for Stroke, *DY* Dynamometer, *VAS* Visual Analogue Scale, *SF-36* Short Form 36, *MS* Multiple Sclerosis, *BBS* Berg Balance Scale, *POMA* Tinetti Performance Oriented Mobility Assessment (b = balance subscale; g = gait subscale), *10MWT* 10 m walking test, *BBA* Brunnel Balance Assessment, *SUS* System Usability Scale, *IMI* Intrinsic Motivation Inventory, *SLB* Single Leg Balance Test, *TUG* Time Up and Go test, *SEQ* Suitability Evaluation Questionnaire

*: significant *p* value (*p* < 0.05) in one or more outcome parameters; £: significant *p* value (*p* < 0.05) in pre- vs posttest; not at follow-up; ES: Effect size

NA: Not applicable

Focusing on the upper limb, in the studies of Levin et al. [2] and Shiri et al. [35], MCS therapy was performed without conventional therapy. Whereas in the study of Sin et al. [3] a combination of conventional therapy with MCS therapy was used. With regard to within group differences, all three studies reported improvement on one or more outcome measures [2, 3, 35]. The improvement was significant in two studies [3, 35], one study only reported effect sizes [2], especially on WMFT, FMA and BBT.

A between group comparison was only possible in two out of the three studies, as Shiri et al. [35] presents a case series design. Levin et al. [2] and Sin et al. [3] reported a positive result in favour of the experimental group. Levin et al. [2] reported a larger effect of training on the WMFT-FAS in the VR group at post-test compared to conventional group. Both post training (with an improvement post training in five out of the six subjects in the experimental group in contrast to three out of six in de control group) and at follow-up (four out of six subjects from the experimental group in contrast to two from the control group), more subjects from the experimental group maintained their improvements at 1 month follow-up as opposed to control group. The other part of the WMFT, i.e. WMFT-TIME, did not show any remarkable effects [2]. Sin et al. [3] reported a significant difference between the experimental group and the control group at post-test for Active Range of Motion (AROM) of all joint movements in shoulder, elbow and wrist, FMA motor function and BBT. At follow-up (i.e. 6 weeks post treatment), the experimental group improved significantly better than the control group for AROM of shoulder and elbow, but not of the wrist, FMA and BBT.

Two studies used and reported the Motor Activity Log, a clinical measurement of perceived performance on ICF activity level [2, 35]. Levin et al. [2] reported that both the frequency (MAL-AOU; Amount of Use) and quality (MAL-QOM; Quality of Movement) of daily arm use was unchanged in both experimental and control group while training was done on both ICF activity and participation level. Shiri et al. [35] reported a significant improvement on the MAL in general after training which was focused on ICF activity level.

Only one study performed a long-term follow-up at 3 months post treatment [35]. Shiri et al. [35] is the only case series selected and performed a long-term follow-up measurement after 3 months post treatment. Levin et al [2] performed a follow-up measurement, but only 1 month after the intervention.

Regarding the lower limb, the training duration and intensity was very different (as shown in Table 3). Although training parameters are divers, all three lower limb studies used the same tool, i.e. Kinect [21, 32, 33].

The study of Llorens et al. [33] focused on the difference between therapy in home-based situation (experimental group) and therapy in rehabilitation centre (control group). It is also the only study of the lower limb that measured at follow-up after 1 month of finishing training in which improvement was retained.

All studies reported a significant effect on the Berg Balance Scale (BBS) with both studies of Llorens et al. showing significant improvement in the experimental as well as in the control group [32, 33]. Lozano-Quilis et al. only reported a significant improvement on the BBS in the experimental group [21]. One study reported a significant improvement on both subscales of the Tinetti Performance Oriented Mobility Assessment, i.e. balance and gait (respectively POMAb and POMAg), and Brunnel Balance Assessment (BBA) in both experimental and control group [33]. Lozano-Quilis et al. [21] further showed significant improvement of the POMAb subscale in both groups. Two studies used the 10-m walking test (10MWT) and both reported significant improvement in the experimental as well as in the control group [21, 32].

The study that assessed the difference in the use of MCS between home and hospital setting did not reveal any significant difference [33]. The other studies showed significant differences between group in favour of the experimental group measured by several outcome measures, i.e. BBS and 10MWT [21, 32]. Other than the BBS and 10MWT, Lozano-Quilis et al. [21] presented significant group by time effect on SLB right foot and significant group effect on the Time “Up and Go” test (TUG).

The only study who included motivation and usability, was the study of Llorens et al. (2015). Regarding motivation, a positive trend was found on the Intrinsic Motivation Inventory (IMI) and System Usability Scale (SUS) after the intervention. In addition, Llorens et al. [33] also found that overall expenses were cheaper in home-based programs in relation to the clinical program.

As all three studies focussing on lower limb use the Kinect, it can be stated that function and/or activity training of the lower limb with Kinect gives significant effect within groups on clinical outcome measures who focus on ICF activity level.

## Discussion

The aim of this study was to investigate 1) which markerless MCS are used as training devices in neurological rehabilitation, 2) how they are applied, 3) in which target population, 4) what the content of the training and 5) efficacy of training with MCS is. In general, the results of this systematic review demonstrate that only a limited number of studies investigated the effects of an upper or lower limb training with (markerless) motion capture systems in people with a neurological disease. The Kinect was mostly used, performing exercises in a virtual environment was mostly applied, stroke was the main target population and the upper limb the most trained body part. Interventions focused mainly on ICF activity level and visual feedback was the most common used form of feedback. A large variety of training parameters, e.g. content, frequency and intensity, was found. None of the studies reported a client-centred task-oriented approach. All studies reported (significant) improvement in upper and lower limb, on one or more ICF levels, mostly in favour of the experimental MCS group.

The Microsoft Kinect was the most used MCS in the selected studies (*n* = 18). Several studies reported advantages of Microsoft Kinect compared to, e.g. robotics or Nintendo Wii. As people using Kinect-based systems do not have to wear sensors or markers on the body or hold them [26], they are found to be more appealing, comfortable, enjoyable and more intuitive to use [21, 27, 33, 39, 40]. It is also commercially available at low cost and can be used at home [26, 27, 33, 39, 40]. These findings are in compliance with two qualitative studies [43, 44]. Knippenberg et al. [43] investigated the opportunities of markerless MCS by assessing the expectations and requirements of therapists and patients towards the use of MCS and Microsoft Kinect in neurological rehabilitation. It was found that the main advantage for patients was the possibility of using the Kinect without assistance of a therapist and to exercise at home [43]. Palacios-Cena et al. [46] reported a high degree of adherence (86.02% completed sessions) and satisfaction (87.4% of the sample was highly satisfied) when performing a Kinect virtual home-exercise program [44]. The main disadvantages of the Microsoft Kinect are the lack of fine movement capturing and restrictions in shoulder joint biomechanical accuracy [27]. Nevertheless, the validity and accuracy of Microsoft Kinect in clinical assessment is strong for postural control and standing balance, as well as reproducibility when analysing planar motion [24–26].

Although the Microsoft Kinect has already been used and implemented in neurological rehabilitation, it is not yet adjusted to the specific needs of neurological rehabilitation [1, 3, 4, 39]. For example standard games used in therapy are motivating, but performance has to be closely monitored as overexertion injuries can occur, and games are not always adapted to individual therapy goals. Furthermore, improvement on in-game scores do not necessarily correlate with actual functional improvement [27]. Also, in the studies included in this review, lots of different forms of applications with MCS are used, which makes comparing the content of training difficult. Hence the lack of consistent evidence on effectiveness regarding functional improvement.

With regard to the included subjects, the most frequently enrolled study population were people with a chronic stroke (*n* = 13). Other studies included subjects with acute and subacute stroke, dementia, MS, Parkinson’s disease or brain injury. This is in relation with the larger prevalence in stroke population than e.g. MS or cerebral palsy [45–47]. However, other patient groups than chronic stroke, such as MS, Parkinson’s Disease, etc., might also benefit a broader implementation of markerless MCS [6, 21, 27, 37, 39, 40, 48]. Especially when developers implement a client-centred task-oriented approach into the system.

The study sample ranged from one subject (case study) [1, 23] to 35 (RCT) [3] and the methodological quality based on Van Tulder Score, was low (8.06 ± 3.67). When looking at the selection of the six studies with at least a Van Tulder score of 9, the sample size was still low, ranging from six (case series) [35] to 35 (RCT) [3] subjects. Hence, no meta-analysis could be performed and conclusions about efficacy should be made with caution.

It is known from the principles of training physiology and motor learning that duration, intensity and content of training are important factors for effectiveness of the training [12, 49, 50]. Despite this knowledge, the wide variety on training duration, intensity and content was remarkable in the selected studies. Due to this wide variety, general assumptions should be made with caution. Regarding duration, the intervention ranged from 2 weeks [23] to 24 weeks [6]. This was probably due to practical considerations such as availability of subjects in rehabilitation centres, and to the fact that most studies considered themselves a pilot and/or feasibility study before larger and longer interventions are planned. Regarding intensity, some studies compared the intervention training with conventional training, a combination of intervention and conventional training with the same intensity and duration, or intervention plus conventional rehabilitation training versus conventional rehabilitation alone. The latter has more rehabilitation time, creating a bias in favour of the new intervention training.

Within the selection of six studies with good methodological quality, it is shown that there is an improvement on most clinical outcome measures after intervention using MCS in combination with virtual reality, with a major focus on ICF activity level. This might be related to task-specific effects of motor learning [14, 51]. Also, the improvement might be due to the gaming-aspect as people tend to be more motivated compared to conventional therapy. But only two studies performed an outcome measurement specifically for motivation: Llorens et al. [33] and Summa et al. (2015) assessed motivation with the IMI [33, 41]. Although the study of Llorens et al. (2015) was an RCT, the experimental group as well as the control group received training with the Kinect. Therefore, the high results in both groups on the IMI, without significant differences, suggests that all patients considered the training with Kinect motivating [33]. This is in accordance with studies regarding virtual reality, who state that when offering virtual rehabilitation exercises, patients are more motivated to perform the exercises [21, 27] and their adherence to the treatment is greater [21].

Regarding the use of assessments, a wide variety of assessment instruments was used, especially in upper limb. The upper limb is considered more complex as opposed to the lower limb [52], which is probably the reason why there were more unique assessments used in the different studies. Also, the fact that two out of three lower limb studies were performed by the same researcher [32, 33], could be of influence. The wide variety of assessment instruments makes comparison between studies more difficult and interpretation of efficacy of training should be made with caution.

It was remarkable that only two (case series) studies had long-term follow-up measurements performed [34, 35]. As a consequence no assumptions can be made to occurrence of true neuroplasticity.

The limited number of studies is likely due to the novelty of using commercially available motion capture systems such as Microsoft Kinect in rehabilitation. In this review, all selected studies were published in the last 5 years, which is in compliance with results of Saposnik et al. [4] and Webster et al. [44]. Saposnik et al. [4] reviewed literature specifically concerning all kinds of VR in stroke rehabilitation, not the use of MCS [4]. While Webster et al. [44] reviewed the applications specifically with Kinect in elderly care and stroke rehabilitation [27].

It also has to be noted that maybe not all relevant articles came forward using the described search strategy. For example the term “Kinect” was included in the search, which provides a selection bias. However, even though the term was explicitly used, other devices were included as well. Another example is the term “rehabilitation”. By using the term “rehabilitation”, other articles such as Colomer et al. [53] and Shih et al. [54] did not came forward using the search strategy because the term “exergaming” was used instead [53, 54].

No client-centred task-oriented approach was used in the selected studies, as they used standardised exercises for all patients. There were only two studies that took the patient’s interest into account by either using images that corresponded with the patient’s interests [6], or by letting the patient choose between a previously set of exercises [31]. While people’s interests are important, a client-centred approach goes much further by actively involving them in selecting their own goals. Implementing a client-centred task-oriented approach might be time consuming, especially with a novel system such as the Microsoft Kinect, but has established its value [10–14]. Therefore, future research is needed with implementation of a client-centred task-oriented approach in combination with markerless MCS such as Microsoft Kinect.

## Conclusion

There are different types of MCS used in neurological rehabilitation, but Microsoft Kinect is mostly used. Most applications target stroke patients and focus on upper limb training. None of the included studies used a client-centred and task-oriented approach.

Because there are a few RCT and CCT and few studies with long-term follow-up, it is difficult to prove efficacy based on the studies included in this review. However, there is a potential to use MCS in combination with a client-centred task-oriented approach. Even more, future technology developments should take up the challenge to combine MCS with the principles of a client-centred task-oriented approach and prove efficacy using RCT with long-term follow-up.

## Additional files

Additional file 1: Figure S1. Flowchart of article selection. (PDF 41 kb)

## Abbreviations

10MWT: 10-meter Walking Test
A: Activity
(A)ROM: (Active) range of motion
BBS: Berg Balance Scale
BBT: Box and Blocks test
CCT: Controlled clinical trial
DC: Descriptive criteria
F: Function
FB: Full body
FMA: Fugl Meyer Assessmenty
ICF: International Classification of Functioning, Disability and Health
IV: Internal validity
IMI: Intrinsic Motivation Inventory
LL: Lower limb
MAL: Motor Activity Log
MCS: Motion Capture Systems
MS: Multiple sclerosis
P: Participation
PD: Parkinson’s disease
POMA: Tinetti Performance Oriented Mobility Assessment
RCT: Randomised controlled trial
SC: Statistical criteria
SCI: Spinal cord injury
SD: Standard deviation
SIS: Stroke Impact Scale
SLB: Single Leg Balance test
SUS: System Usability Scale
TBI: Traumatic brain injury
UL: Upper limb
VR: Virtual Reality
WMFT: Wolf Motor Function Test

## References

[1] Brokaw EB, Eckel E, Brewer BR: Usability evaluation of a kinematics focused Kinect therapy program for individuals with stroke. Technol Health Care. 2014;23:143–151. 10.3233/THC-140880 25425584

[2] Levin MF, Snir O, Liebermann DG, Weingarden H, Weiss PL (2012). Virtual reality versus conventional treatment of reaching ability in chronic stroke: clinical feasibility study. Neurol Ther.

[3] Sin H, Lee G (2013). Additional virtual reality training using Xbox Kinect in stroke survivors with hemiplegia. Am J Phys Med Rehabil.

[4] Saposnik G, Levin M (2011). Virtual reality in stroke rehabilitation a meta-analysis and implications for clinicians. Stroke.

[5] Llorens R, Alcaniz M, Colomer C, Navarro MD (2012). Balance recovery through virtual stepping exercises using Kinect skeleton tracking: a follow-up study with chronic stroke patients. Stud Health Technol Inform.

[6] Jaume-i-Capo A, Martinez-Bueso P, Moya-Alcover B, Varona J (2014). Interactive rehabilitation system for improvement of balance therapies in people with cerebral palsy. IEEE Trans Neural Syst Rehabil Eng.

[7] Kwakkel G, van Peppen R, Wagenaar RC, Wood Dauphinee S, Richards C, Ashburn A, Miller K, Lincoln N, Partridge C, Wellwood I, Langhorne P (2004). Effects of augmented exercise therapy time after stroke: a meta-analysis. Stroke.

[8] Rietberg MB, Brooks D, Uitdehaag BM, Kwakkel G: Exercise therapy for multiple sclerosis. Cochrane Database Syst Rev. 2005;3:Cd003980. 10.1002/14651858.CD003980.pub2 PMC6485797 15674920

[9] Smidt N, de Vet HC, Bouter LM, Dekker J, Arendzen JH, de Bie RA, Bierma-Zeinstra SM, Helders PJ, Keus SH, Kwakkel G (2005). Effectiveness of exercise therapy: a best-evidence summary of systematic reviews. Aust J Physiother.

[10] Spooren AI, Janssen-Potten YJ, Kerckhofs E, Bongers HM, Seelen HA (2011). ToCUEST: a task-oriented client-centered training module to improve upper extremity skilled performance in cervical spinal cord-injured persons. Spinal Cord.

[11] Timmermans AAA, Geers RPJ, Franck JA, Dobbelsteijn P, Spooren AIF, Kingma H, Seelen HAM: T-TOAT: A method of task-oriented arm training for stroke patients suitable for implementation of exercises in rehabilitation technology. In Rehabilitation Robotics, 2009 ICORR 2009 IEEE International Conference on; 23–26 June 2009. 2009: 98–102.

[12] Timmermans A, Spooren A, Kingma H, Seelen H (2010). Influence of task-oriented training content on skilled arm-hand performance in stroke: a systematic review. Neurorehabil Neural Repair.

[13] Van Peppen R, Kwakkel G, Wood-Dauphinee S, Hendriks H, Van der Wees P, Dekker J (2004). The impact of physical therapy on functional outcomes after stroke: what's the evidence?. Clin Rehabil.

[14] Spooren AI, Timmermans AA, Seelen HA (2012). Motor training programs of arm and hand in patients with MS according to different levels of the ICF: a systematic review. BMC Neurol.

[15] Prange GB, Jannink MJ, Groothuis-Oudshoorn CG, Hermens HJ, Ijzerman MJ (2006). Systematic review of the effect of robot-aided therapy on recovery of the hemiparetic arm after stroke. J Rehabil Res Dev.

[16] Feys P, Coninx K, Kerkhofs L, De Weyer T, Truyens V, Maris A, Lamers I (2015). Robot-supported upper limb training in a virtual learning environment: a pilot randomized controlled trial in persons with MS. J Neuroeng Rehabil.

[17] Gijbels D, Lamers I, Kerkhofs L, Alders G, Knippenberg E, Feys P (2011). The Armeo spring as training tool to improve upper limb functionality in multiple sclerosis: a pilot study. J Neuroeng Rehabil.

[18] Fasoli SE, Krebs HI, Stein J, Frontera WR, Hogan N (2003). Effects of robotic therapy on motor impairment and recovery in chronic stroke. Arch Phys Med Rehabil.

[19] Timmermans AA, Lemmens RJ, Monfrance M, Geers RP, Bakx W, Smeets RJ, Seelen HA (2014). Effects of task-oriented robot training on arm function, activity, and quality of life in chronic stroke patients: a randomized controlled trial. J Neuroeng Rehabil.

[20] Vanmulken DA, Spooren AI, Bongers HM, Seelen HA (2015). Robot-assisted task-oriented upper extremity skill training in cervical spinal cord injury: a feasibility study. Spinal Cord.

[21] Lozano-Quilis JA, Gil-Gomez H, Gil-Gomez JA, Albiol-Perez S, Palacios-Navarro G, Fardoun HM, Mashat AS (2014). Virtual rehabilitation for multiple sclerosis using a kinect-based system: randomized controlled trial. JMIR Serious Games.

[22] Adamovich SV, Fluet GG, Tunik E, Merians AS (2009). Sensorimotor training in virtual reality: a review. Neuro Rehabilitation.

[23] Pastor I, Hayes HA, Bamberg SJ (2012). A feasibility study of an upper limb rehabilitation system using Kinect and computer games. Conf Proc IEEE Eng Med Biol Soc.

[24] Clark RA, Pua YH, Fortin K, Ritchie C, Webster KE, Denehy L, Bryant AL (2012). Validity of the Microsoft Kinect for assessment of postural control. Gait Posture.

[25] Clark RA, Pua YH, Oliveira CC, Bower KJ, Thilarajah S, McGaw R, Hasanki K, Mentiplay BF (2015). Reliability and concurrent validity of the Microsoft Xbox one Kinect for assessment of standing balance and postural control. Gait Posture.

[26] Bonnechere B, Jansen B, Salvia P, Bouzahouene H, Omelina L, Moiseev F, Sholukha V, Cornelis J, Rooze M, Van Sint JS (2014). Validity and reliability of the Kinect within functional assessment activities: comparison with standard stereophotogrammetry. Gait Posture.

[27] Webster D, Celik O (2014). Systematic review of Kinect applications in elderly care and stroke rehabilitation. J Neuroeng Rehabil.

[28] Van Tulder M, Furlan A, Bombardier C, Bouter L, Group EBotCCBR (2003). Updated method guidelines for systematic reviews in the Cochrane collaboration back review group. Spine.

[29] Berry KJ, Mielke PW (1988). A generalization of Cohen's kappa agreement measure to interval measurement and multiple raters. Educ Psychol Meas.

[30] Banerjee M, Capozzoli M, McSweeney L, Sinha D. Beyond kappa: a review of interrater agreement measures. Can J Stat /La Revue Canadienne de Statistique. 1999:3–23.

[31] Lee G (2013). Effects of training using video games on the muscle strength, muscle tone, and activities of daily living of chronic stroke patients. J Phys Ther Sci.

[32] Lloréns R, Gil-Gómez J-A, Alcañiz M, Colomer C, Noé E (2015). Improvement in balance using a virtual reality-based stepping exercise: a randomized controlled trial involving individuals with chronic stroke. Clin Rehabil.

[33] Lloréns R, Noé E, Colomer C, Alcañiz M (2015). Effectiveness, usability, and cost-benefit of a virtual reality–based Telerehabilitation program for balance recovery after stroke: a randomized controlled trial. Arch Phys Med Rehabil.

[34] Bao X, Mao Y, Lin Q, Qiu Y, Chen S, Li L, Cates RS, Zhou S, Huang D (2013). Mechanism of Kinect-based virtual reality training for motor functional recovery of upper limbs after subacute stroke. Neural Regen Res.

[35] Shiri S, Feintuch U, Lorber-Haddad A, Moreh E, Twito D, Tuchner-Arieli M, Meiner Z (2012). Novel virtual reality system integrating online self-face viewing and mirror visual feedback for stroke rehabilitation: rationale and feasibility. Top Stroke Rehabil.

[36] Chang YJ, Chen SF, Chuang AF (2011). A gesture recognition system to transition autonomously through vocational tasks for individuals with cognitive impairments. Res Dev Disabil.

[37] Pompeu JE, Arduini LA, Botelho AR, Fonseca MB, Pompeu SM, Torriani-Pasin C, Deutsch JE (2014). Feasibility, safety and outcomes of playing Kinect adventures! For people with Parkinson's disease: a pilot study. Physiotherapy.

[38] Ustinova K, Perkins J, Leonard W, Ingersoll C, Hausebeck C: Virtual reality game-based therapy for persons with TBI: A pilot study. In Virtual Rehabilitation (ICVR), 2013 International Conference on. IEEE; 2013: 87–93.

[39] Palacios-Navarro G, Garcia-Magarino I, Ramos-Lorente P (2015). A Kinect-based system for lower limb rehabilitation in Parkinson's disease patients: a pilot study. J Med Syst.

[40] Summa S, Basteris A, Betti E, Sanguineti V (2015). Adaptive training with full-body movements to reduce bradykinesia in persons with Parkinson's disease: a pilot study. J Neuroeng Rehabil.

[41] Summa S, Pierella C, Giannoni P, Sciacchitano A, Iacovelli S, Farshchiansadegh A, Mussa-Ivaldi FA, Casadio M (2015). A body-machine interface for training selective pelvis movements in stroke survivors: a pilot study. Conf Proc IEEE Eng Med Biol Soc.

[42] GestureXtreme (r) Immersive Virtual Gaming System [http://www.gesturetek.com/gesturextreme/introduction.php]. Accessed 3 Dec 2015.

[43] Knippenberg E, Spooren A: Opportunities of markerless motion detection systems for use in neurological rehabilitation: a qualitative study on patient and therapist perspective. Austin J Robot Autom. 2016;3:1-5.

[44] Palacios-Cena D, Ortiz-Gutierrez RM, Buesa-Estellez A, Galan-Del-Rio F, Cachon PJ, Martinez-Piedrola R, Velarde-Garcia J, Cano-De-la-Cuerda R (2016). Multiple sclerosis patients' experiences in relation to the impact of the kinect virtual home-exercise programme: a qualitative study. Eur J Phys Rehabil Med.

[45] Heart Disease, Stroke and Research Statistics At-A-Glance. [https://www.heart.org/idc/groups/ahamah-public/@wcm/@sop/@smd/documents/downloadable/ucm_480086.pdf]. Accessed 27 June 2016.

[46] Prevalence of Cerebral Palsy [http://www.cerebralpalsy.org/about-cerebral-palsy/prevalence-and-incidence]. Accessed 27 June 2016.

[47] Multiple Sclerosis by the numbers: Facts, Statistics, and you. [http://www.healthline.com/health/multiple-sclerosis/facts-statistics-infographic]. Accessed 27 June 2016.

[48] Ustinova KI, Perkins J, Leonard WA, Hausbeck CJ (2014). Virtual reality game-based therapy for treatment of postural and co-ordination abnormalities secondary to TBI: a pilot study. Brain Inj.

[49] Langhorne P, Coupar F, Pollock A (2009). Motor recovery after stroke: a systematic review. Lancet Neurol.

[50] Maas E, Robin DA, Hula SNA, Freedman SE, Wulf G, Ballard KJ, Schmidt RA (2008). Principles of motor learning in treatment of motor speech disorders. Am J Speech Lang Pathol.

[51] Timmermans AA, Seelen HA, Willmann RD, Kingma H (2009). Technology-assisted training of arm-hand skills in stroke: concepts on reacquisition of motor control and therapist guidelines for rehabilitation technology design. J Neuroeng Rehabil.

[52] Rau G, Disselhorst-Klug C, Schmidt R (2000). Movement biomechanics goes upwards: from the leg to the arm. J Biomech.

[53] Colomer C, Llorens R, Noé E, Alcañiz M (2016). Effect of a mixed reality-based intervention on arm, hand, and finger function on chronic stroke. J Neuroeng Rehabil.

[54] Shih M-C, Wang R-Y, Cheng S-J, Yang Y-R (2016). Effects of a balance-based exergaming intervention using the Kinect sensor on posture stability in individuals with Parkinson’s disease: a single-blinded randomized controlled trial. J Neuroeng Rehabil.

