# Supplementary material for: Markerless motion capture systems as training device in neurological rehabilitation: a systematic review of their use, application, target population and efficacy
Source: J Neuroeng Rehabil. 2017 Jun 24;14:61. doi: 10.1186/s12984-017-0270-x (PMC5482943; doi:10.1186/s12984-017-0270-x)
Supplement: Supplementary file 1 — Flowchart of article selection. (PDF 41 kb) [file 12984_2017_270_MOESM1_ESM.pdf]

PRISMA 2009 Flow Diagram

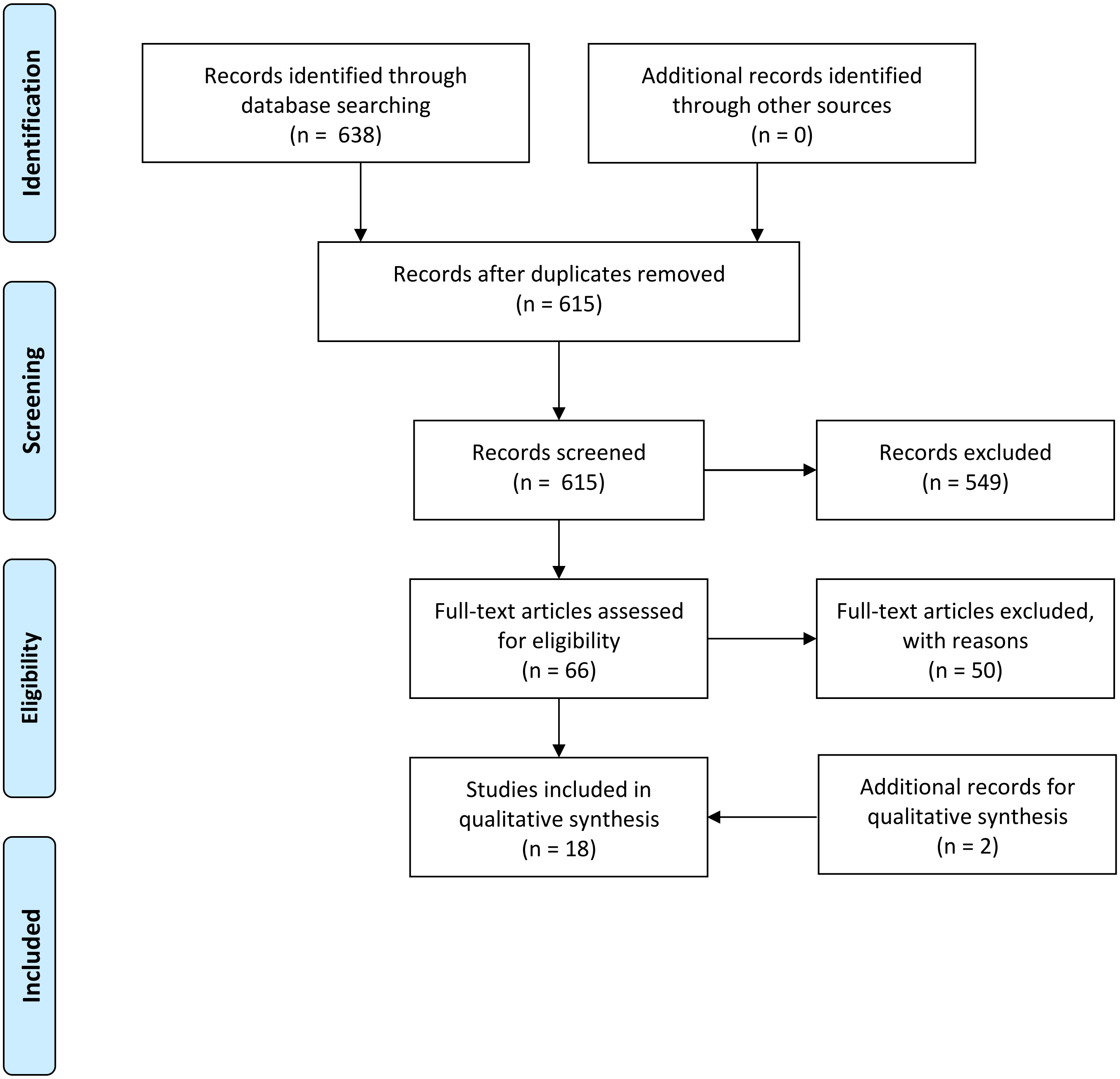

From: Moher D, Liberati A, Tetzlaff J, Altman DG, The PRISMA Group (2009). *P*referred *R*eporting *I*tems for *S*ystematic Reviews and *M*eta-*A*nalyses: The PRISMA Statement. PLoS Med 6(6): e1000097. doi:10.1371/journal.pmed1000097

For more information, visit www.prisma-statement.org.
